# Factors Affecting Physical Activity Adherence in Male Office Workers Based on Self-Determination Theory: The Mediating Effects of Psychological Need Satisfaction and Autonomous Motivation

**DOI:** 10.3390/healthcare13151852

**Published:** 2025-07-30

**Authors:** Sangmi Han, Yeongmi Ha

**Affiliations:** 1 Department of Nursing, Gyeongnam Geochang University, Geochang 50147, Gyeongnam, Republic of Korea; y2k2509@nate.com; 2 College of Nursing and Sustainable Health Research Institute, Gyeongsang National University, Jinju 52727, Gyeongnam, Republic of Korea

**Keywords:** office workers, physical activity adherence, psychological need satisfaction, motivation, self-determination theory

## Abstract

**Background/Objectives:** Despite the health benefit of regular physical activity, many adults often discontinue it within 3–6 months due to various obstacles. The purpose of this study was to investigate factors affecting physical activity adherence of male office workers based on self-determination theory by constructing a structural equation model. **Methods:** In total, 257 full-time male office workers who engage in regular physical activity participated. The participants from 15 companies completed a survey asking about perceived physical activity barriers, autonomy support, psychological need satisfaction in physical activity, autonomous motivation, and physical activity adherence. Data analysis was performed using the SPSS 28.0 and the AMOS 26.0 programs to verify the fit of the hypothetical model and identify the direct and indirect effects of variables on physical activity adherence for male office workers. **Results:** As a result, the path significance test results for the hypothetical model showed that five of the nine paths were significant. The results show that psychological need satisfaction in physical activity and autonomous motivation were significant variables that had a direct effect on physical activity adherence, while autonomy support from significant others and perceived physical activity barriers had a significant indirect effect through psychological need satisfaction and autonomous motivation, explaining 62.0%. **Conclusions:** Based on these findings, it is recommended to implement customized workplace-specific physical activity interventions to enhance autonomous motivation and the autonomy, competence, and relatedness aspects of psychological need satisfaction in physical activity.

## 1. Introduction

### 1.1. Background

According to the 2018 Physical Activity Guidelines for Americans [1] and 2023 Physical Activity Guidelines for Koreans [2], adults aged 18 through 64 years should do at least 150–300 min a week of moderate-intensity, or 75–150 min a week of vigorous-intensity aerobic physical activity, or an equivalent combination of moderate- and vigorous-intensity aerobic activity. Office workers are known to be at risk of insufficient physical activity due to their work characteristics, which require them to spend at least seven hours a day sitting [3]. The lack of physical activity caused by their job characteristics contributes to many adverse health outcomes, including all-cause mortality and the risk of developing chronic diseases such as stroke, coronary heart diseases, diabetes, and metabolic syndrome [1,4]. Despite the risks of physical inactivity, many adults often find it difficult to begin moderate–vigorous physical activity (MVPA) or tend to discontinue it within 3–6 months due to various barriers [5,6]. In particular, regarding its health benefits, physical activity adherence is recommended on three or more days a week, and adults should minimize prolonged sitting time throughout the day [1,2].

The definition of physical activity adherence varies among scholars, but it generally refers to the consistent practice of regular physical activity as an integral part of one’s life, and it includes factors such as frequency, intensity, duration, and type of activity [4]. Most previous studies have focused on physical activity adherence among patients with various chronic diseases [7,8], while research examining the physical activity adherence of general adults is limited. According to a systematic review on physical activity adherence among patients prescribing exercise therapy, the factors that significantly affected physical activity adherence were support from family or health professionals, enjoyment of physical activity, and personal wellness goals [8]. Since long-term physical activity adherence clearly has positive effects on health, the factors affecting the physical activity adherence of office workers should be explored.

Self-determination theory, well known as explaining motivational processes for physical activity, has demonstrated how regular physical activity is initiated and maintained in the long-term through motivational processes [9,10]. Behavioral motivation is the most powerful driving force within an individual that impels them to pursue their positive behavioral changes [11]. A growing body of research and systematic reviews have been conducted on behavioral motivation toward physical activity—i.e., why some people show an enduring desire to pursue their activity, whereas others lose interest [12,13]. Behind the motivational process for physical activity, applying self-determination theory, is the premise that people are inherently active in their pursuit to satisfy three basic psychological needs: the need to feel a sense of engagement during physical activity (autonomy); a belief in one’s ability to perform the physical activity well (competence); and feeling a meaningful connection with others participating in the same physical activity (relatedness) [10,12]. The fulfillment of these three basic needs for physical activity determines individuals’ persistence toward engaging in intrinsically motivated activity behaviors, thereby leading to long-term physical activity adherence [13]. Pelletier et al. [14] developed a sports motivation scale based on self-determination theory, which allows for a precise assessment of an individual’s intrinsic and extrinsic motivation to participate in sports and regular physical activity. Individuals with high intrinsic motivation for physical activity tend to find MVPA interesting and enjoyable, which drives them to continue engaging in it voluntarily over a long period [4,9,11].

Perceived barriers to physical activity can be an important factor that determines whether individuals continue or discontinue physical activity [15]. Adults generally discontinue regular physical activity for reasons such as ‘time restriction,’ ‘lack of knowledge or exercise skills,’ ‘poor accessibility to physical activity facilities,’ and ‘burden of expenses for MVPA’ [8,16]. According to previous research about barriers to reduce sedentary behaviors and increase physical activity of office workers, major barriers were personal factors such as lack of motivation, job characteristics such as lack of time, cost, distance, and job stress, or organizational factors such as poor accessibility [17,18]. These barriers are known to reduce confidence and decrease intrinsic motivation for physical activity, ultimately making physical activity adherence more difficult [8]. Several suggestions have emerged to decrease the sedentary behaviors and improve the physical activity of office workers such as that more support from companies, cultivating health supporting organizational culture, and high accessibility to exercise facilities in companies should be provided [18]. Therefore, this study aimed to identify predictors of physical activity adherence among office workers based on self-determination theory [19]. First, the purpose of this study is to build a hypothetical model for the physical activity adherence of male office workers based on self-determination theory and literature reviews. Second, it is verified whether the relationship between the hypothetical model and the actual data is suitable for the model of self-determination theory. Third, the study identifies the factors that affect the physical activity adherence of male office workers and identifies direct and indirect effects and total effects.

### 1.2. Conceptual Framework

**Figure 1:**
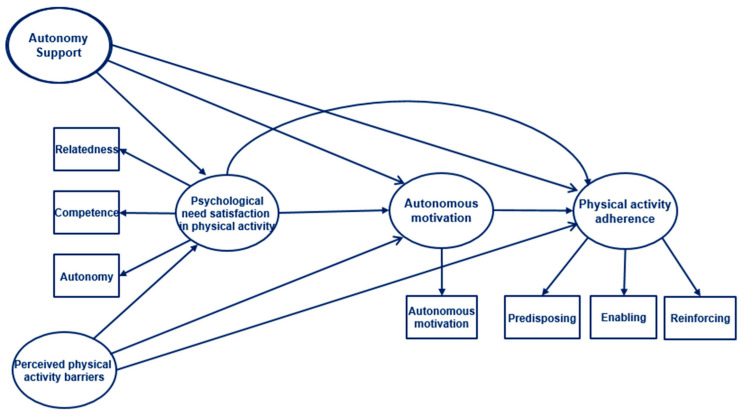
Hypothetical model.

The conceptual framework of this study was established based on self-determination theory [19] and literature reviews related to physical activity for adults. When three basic psychological needs for autonomy, competence, and relatedness in physical activity are fulfilled, people’s intrinsic motivation is enhanced, leading them to actively engage in physical activity adherence. Self-determination theory and previous studies demonstrate autonomous motivation as a significant influencing factor for physical activity adherence [10,13]. In the hypothetical model of this study, exogenous variables such as perceived physical activity barriers and autonomy support from significant others affect the endogenous variables, such as psychological need satisfaction in physical activity and autonomous motivation; these factors, in turn, established both direct and indirect pathways toward physical activity adherence of office workers (Figure 1). The hypotheses in this study are as follows:

#### 1.2.1. Hypothesis with Psychological Need Satisfaction in Physical Activity as an Endogenous Variable

Autonomy support (Hypothesis 1, H1) and perceived physical activity barriers (Hypothesis 2, H2) are expected to be positively and significantly associated with psychological need satisfaction in physical activity.

#### 1.2.2. Hypothesis with Autonomous Motivation as an Endogenous Variable

**Hypothesis 3 (H3).**  *Autonomy support is expected to be positively and significantly associated with autonomous motivation.*

**Hypothesis 4 (H4).**  *Perceived physical activity barriers are expected to be positively and significantly associated with autonomous motivation.*

**Hypothesis 5 (H5).**  *Psychological need satisfaction in physical activity is expected to be positively and significantly associated with autonomous motivation (H5a), and psychological need satisfaction in physical activity is hypothesized to act as a mediator in the pathways between autonomy support, perceived physical activity barriers, and autonomous motivation (H5b).*

#### 1.2.3. Hypothesis with Physical Activity Adherence as an Endogenous Variable

**Hypothesis 6 (H6).**  *Autonomy support is expected to be positively and significantly associated with physical activity adherence.*

**Hypothesis 7 (H7).**  *Perceived physical activity barriers are expected to be positively and significantly associated with physical activity adherence.*

**Hypothesis 8 (H8).**  *Psychological need satisfaction in physical activity is expected to be positively and significantly associated with physical activity adherence.*

**Hypothesis 9 (H9).**  *Autonomous motivation is expected to be positively and significantly associated with physical activity adherence (H9a); autonomous motivation is hypothesized to act as a mediator in the pathways between psychological need satisfaction in physical activity and physical activity adherence (H9b); and autonomous motivation is hypothesized to act as a mediator in the pathways between autonomy support, perceived physical activity barriers, and physical activity adherence (H9c).*

## 2. Methods

### 2.1. Participants

This study conveniently sampled male office workers working in the banking sector in a city in Korea. The inclusion criteria for the participants were as follows: (1) office workers aged between 19 and 60; (2) full-time workers working 40 h per week; and (3) individuals engaging in 150–300 min of moderate aerobic physical activity or 75–150 min of vigorous aerobic physical activity per week. The exclusion criteria were determined based on literature reviews that explore whether accessibility, preference for physical activity adherence, basic psychological needs, and activity barriers and facilitators have a moderating effect depending on gender [20,21,22,23,24]. First, previous studies demonstrated that men and women differ in their response to autonomy support, with men showing stronger associations between autonomy support and intrinsic motivation for physical activity [17,21,22,23]. Other research has shown that the relative importance of the three basic psychological needs (autonomy, competence, and relatedness) varies significantly between genders, with men typically showing higher sensitivity to competence-related factors while women place greater emphasis on relatedness aspects [18,21]. Second, workplace-based physical activity research has revealed distinct gender-specific barriers and facilitators [24]. Male office workers face unique challenges including workplace culture expectations around masculinity and physical performance, different social support structures, and varying responses to workplace interventions [24]. Hence, women were excluded from this study due to gender differences in physical activity adherence. Temporary or part-time workers were also excluded since their working hours and environments differ from those of full-time workers.

The sample size was determined according to current best practices for structural equation modeling [25,26,27]. Following recent recommendations [25,26,27], our sample of 257 participants meets current guidelines: (1) it exceeds the minimum 200 cases recommended for complex SEM models; (2) it satisfies the updated 10:1 participant-to-parameter ratio with 25 parameters estimated in our model; and (3) it provides adequate power (>0.80) for detecting medium effect sizes (β = 0.30) at α = 0.05 based on Monte Carlo simulations. The final 257 cases in the dataset were used for analysis, after excluding 18 cases with missing data.

### 2.2. Measurements

The general characteristics of the participants included age, marital status, educational level, having a chronic disease, subjective health status, total working experience, daily amount of MVPA, weekly frequency of MVPA, years of MVPA, and physical activity adherence. The survey comprised a total of 66 items.

#### 2.2.1. Physical Activity Adherence

Physical activity adherence was measured using the Physical Activity Adherence Questionnaire [4]. The English version of this instrument was translated into Korean using a double translation process, in which two bilingual individuals translated the English version into Korean and back-translated it into English, after which the finalized Korean version of the questionnaire was used for this study [28]. This instrument comprises three subdomains: predisposing, enabling, and reinforcing factors. Each subdomain has 4 items, for a total of 12 items. Examples of items include “I am very knowledgeable about physical activity (predisposing factor), I know how to plan my own physical activity (enabling factor), I have the support of my family for doing my regular physical activity (reinforcing factor).” Each item is rated on a 3-point Likert scale, with 1 point for “Not true,” 2 points for “Somewhat true,” and 3 points for “Very true,” with higher scores indicating higher physical activity adherence. A score of 33–36 points indicates “Adherence likely,” 27–32 points indicates “Adherence possible,” and below 27 points indicates “Adherence unlikely.” In this study, the overall Cronbach’s α was 0.85, the Cronbach’s α of the predisposing factor was 0.74, the enabling factor was 0.68, and the reinforcing factor was 0.70.

#### 2.2.2. Autonomous Motivation

Autonomous motivation was measured using the Behavioral Regulation in Exercise Questionnaire-2 (BREQ-2) developed by Wilson and Rogers [29]. A previous study on physical activity by Ha and Han [30] translated the BREQ-2 into Korean and conducted tests for cultural appropriateness, with a Cronbach’s α value of 0.89. The BREQ-2 consists of 19 items, but this study used 7 items measuring autonomous motivation including identified regulation and intrinsic regulation, which refers to actively participating in physical activity for enjoyment, satisfaction, and interest. Examples of items include “I take part in exercise because my friends/family/partner say I should, I enjoy my exercise sessions.” The items are rated on a 5-point Likert scale, with 0 point for “Not true for me,” and 4 points for “very true for me,” with higher scores indicating higher autonomous motivation for physical activity. In this study, the overall Cronbach’s α was 0.84.

#### 2.2.3. Psychological Need Satisfaction in Physical Activity

Psychological need satisfaction in physical activity (autonomy, competence, and relatedness) was measured using the Psychological Need Satisfaction in Exercise (PNSE) questionnaire developed by Wilson et al. [31]. Previous study on physical activity by Ha and Han [30] translated the PNSE into Korean and conducted cultural appropriateness testing with a Cronbach’s α value of 0.92~0.95. The PNSE comprises 18 items, with 6 items each for perceived autonomy, perceived competence, and perceived relatedness. Examples of items include “I feel free to exercise in my own way (perceived autonomy), I feel that I am able to complete exercises that are personally challenging (perceived competence), I feel connected to the people who I interact with while we exercise together (perceived relatedness).” The items are rated on a 6-point Likert scale, with higher scores indicating higher autonomy, competence, and relatedness. In this study, the overall Cronbach’s α of psychological need satisfaction in physical activity was 0.94, the perceived autonomy was 0.87, the perceived competence was 0.95, and the perceived relatedness was 0.94.

#### 2.2.4. Autonomy Support

Autonomy support was measured using the Important Other Climate Questionnaire (IOCQ) developed by Williams et al. [32]. A previous study using self-determination theory by Seo and Choi [33] translated the IOCQ into Korean and conducted cultural appropriateness testing with a Cronbach’s α value of 0.87. Examples of items include “I feel that my important others have provided me with choices and options about physical activity, My important others encourage me to ask questions about physical activity.” The IOCQ comprises 6 items rated on a 5-point Likert scale, with higher scores indicating higher autonomy support from significant others. The Cronbach’s α in this study was 0.85.

#### 2.2.5. Perceived Physical Activity Barriers

The Exercise Benefits/Barriers Scale [34] comprises 14 items on perceived exercise barriers and 14 items on perceived exercise benefits. A previous study on physical activity by Seo and Ha [35] translated the EBBS into Korean and conducted cultural appropriateness testing with a Cronbach’s α value of 0.89. This study used only the perceived exercise barriers scale from the EBBS. Examples of items include “Physical activity takes too much of my time, Physical activity tires me” The instrument rated on a 4-point Likert scale, with higher scores indicating greater perceived barriers to physical activity. The Cronbach’s α in this study was 0.86.

### 2.3. Data Collection

After obtaining Institutional Review Board (No: GIRB-A20-Y-0009) approval, data were collected from male office workers working at 15 banks in one city in Korea. The researcher contacted the human resources departments of each bank in advance by phone to explain the purpose and procedures of the study and to ask their permission. To recruit participants, the researcher visited the workplaces in person. Office workers were recruited to participate in the survey near the cafeterias or dining areas they use. The purpose and procedures of the study were explained to the male office workers and, after obtaining voluntary consent from those who voluntarily agreed to participate and met the inclusion and exclusion criteria of this study, written consent was obtained before distributing the paper-based questionnaires. The survey took 20 min to complete, and a small gift was given after completion.

### 2.4. Data Analysis

The data were analyzed as follows using SPSS 28.0 and AMOS 26.0 software. First, the general characteristics of the participants and measured variables were analyzed using descriptive statistics. Skewness and kurtosis were checked to verify the normality, and tolerance limits and variance inflation factors (VIFs) were calculated for multicollinearity. The general characteristics and physical activity-related characteristics were analyzed by frequency, percentage, mean, and standard deviation. The differences in physical activity adherence by the general characteristics and physical activity-related characteristics of participants were evaluated using *t*-tests, ANOVA, and the Scheffé post hoc test. Second, confirmatory factor analysis was conducted to examine the validity and reliability of the latent variables and test the fit of the measurement model. Third, absolute fit indices such as χ^2^, χ^2^/df, root mean square residual (RMR), root mean squared error of approximation (RMSEA), goodness-of-fit index (GFI), and adjusted goodness-of-fit index (AGFI) were calculated to verify the fit of the hypothetical model. The incremental fit indices used included the normed fit index (NFI), the Tucker–Lewis index (TLI), and the comparative fit index (CFI). Acceptable values as goodness of fit indicators are an RMR of less than 0.05, an RMSEA of less than 0.08, a GFI of 0.90 or higher, an AGFI of 0.90 or higher, an NFI of 0.90 or higher, a TLI of 0.90 or higher, and a CFI of 0.95 or higher [36]. Fourth, standardized regression weights, critical ratios, and *p*-values were utilized to analyze the significance of the path coefficients in the hypothetical model, and the explanatory power of the endogenous variables was measured using squared multiple correlations (SMCs). Moreover, the significance of the direct, indirect, and total effects was examined using Bootstrap Maximum Likelihood estimation with 5000 bootstrapping resamples (AMOS 26.0), and the effects were tested at a 95% confidence interval [37].

## 3. Results

### 3.1. Differences in Physical Activity Adherence by General Characteristics of Participants

The participants’ age was 40.85 years. Of the participants, 68.5% were married, and 83.3% were at least college graduates. Over two-thirds of the participants (77.0%) reported no chronic diseases and perceived their health status as moderate or good. Total working experience was 12.39 years. The amount of daily MVPA was 85.21 min, the weekly MVPA frequency was 3.75 days, and average years of MVPA duration was 4.17 years. In terms of physical activity adherence, 44.7% of the participants were classified as “adherence unlikely” (below 27 points), 44.0% as “adherence possible” (27–32 points), and 11.3% as “adherence likely” (33–36 points).

Significant differences in physical activity adherence according to the general characteristics of the participants were found in education level (F = 5.34, *p* = 0.005), subjective health status (F = 7.83, *p* = 0.001), weekly frequency of MVPA (t = −2.52, *p* = 0.012), and years of MVPA duration (F = 11.87, *p* < 0.001). In terms of educational level, significant differences in physical activity adherence were found between those with associate’s degree and those with bachelor’s degree. For subjective health status, significant differences were observed between those who perceived their health as good and those who perceived their health as poor. In terms of MVPA, there were significant differences between more than 4 days of weekly MVPA and 3 days of weekly MVPA. In addition, significant differences in physical activity adherence were observed between more than 5 years of MVPA and less than 3 years of MVPA (Table 1).

### 3.2. Descriptive Statistics and Convergent Validity of Variables

The perceived physical activity barriers score was 1.69 ± 0.38 (range 1–4), autonomy support was 3.71 ± 0.62 (range 1–5), and psychological need satisfaction in physical activity was 4.64 ± 0.74 (range 1–6), with the subdomains of autonomy, competence, and relatedness obtaining scores of 4.88 ± 0.75, 4.40 ± 1.01, and 4.65 ± 0.87, respectively. The autonomous motivation score was 3.20 ± 0.56 (range 0–4), and physical activity adherence was 27.19 ± 4.39 (range 12–36).

Regarding the normality of the research variables, skewness ranged from −1.03 to 0.02 and kurtosis ranged from −0.75 to 2.08, meeting the assumption of normal distribution. Multicollinearity was not found among the measurement variables of the hypothetical model. Regarding convergent validity to test the validity of the latent variables, the average variance extracted (AVE) ranged from 0.63 to 0.87 and construct reliability ranged from 0.84 to 0.95; hence, the convergent validity of the variables was established (Table 2).

### 3.3. Direct, Indirect, and Total Effects of the Hypothetical Model

**Figure 2:**
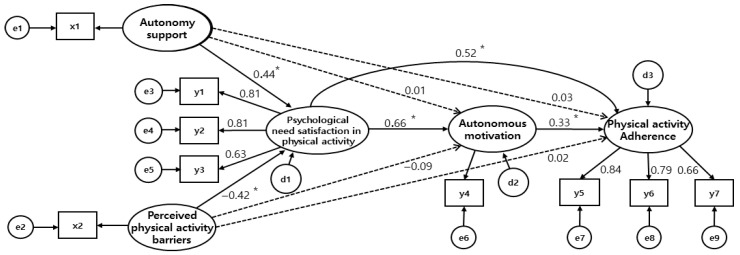
Path diagram for the final model. x1 = Autonomy support; x2 = Perceived physical activity barriers; y1 = Relatedness; y2 = Competence; y3 = Autonomy; y4 = Autonomous motivations; y5 = Predisposing; y6 = Enabling; y7 = Reinforcing (Goodness-of fit-indicators: χ^2^ = 51.53, *p* < 0.001, χ^2^/df = 2.577, RMR = 0.01, RMSEA = 0.07, GFI = 0.96, AGFI = 0.90, NFI = 0.95, TLI = 0.94, CFI = 0.97). * *p* < 0.01.

The goodness-of-fit test results of the hypothetical model showed that both absolute fit indices (RMR, RMSEA, GFI, and AGFI) and incremental fit indices (NFI, TLI, and CFI) met the recommended levels, indicating an acceptable model fit. The path significance test results for the hypothetical model showed that five out of nine paths were significant (Table 3, Figure 2). For psychological need satisfaction in physical activity, direct effects were observed from autonomy support (β = 0.44, *p* < 0.001) and perceived physical activity barriers (β = −0.42, *p* < 0.001). For autonomous motivation, direct effects from autonomy support (β = 0.01, *p* = 0.861) and perceived physical activity barriers (β = −0.09, *p* = 0.115) were not significant. Psychological need satisfaction in physical activity had a direct effect on autonomous motivation (β = 0.66, *p* < 0.001). For physical activity adherence, the direct effect (β = 0.03, *p* = 0.695) of autonomy support was not significant. The direct effect of perceived physical activity barriers (β = 0.02, *p* = 0.723) was not significant. The direct effect (β = 0.52, *p* < 0.001), indirect effect (β = 0.22, *p* = 0.004), and total effect (β = 0.74, *p* = 0.005) of psychological need satisfaction in physical activity were significant, while the direct effect of autonomous motivation (β = 0.33, *p* < 0.001) was significant.

## 4. Discussion

The increase in office jobs due to the decline of labor-intensive jobs in South Korea, along with the inherent characteristics of office work, has raised the risk of chronic diseases among office workers. Hence, it is more important than ever to understand physical activity adherence in office workers. The participants in this study were male office workers with an average age of 40.85 years, and 44.7% of them fell into the category of “physical activity adherence unlikely.” This raises the need for greater attention to improving physical activity adherence among office workers. It is crucial to create health promoting environments that support physical activity adherence among office workers working in sedentary jobs for 8 h a day, 5 days a week [3]. For instance, it may be helpful to create an environment that encourages office workers to use the stairs instead of elevators, develop a workplace physical activity program during lunch or break time, change the office environment to increase physical activity within the office, create an environment where workers can work standing up, and form walking paths to encourage workers to walk around the workplace [38]. Additionally, our findings specifically indicate that higher education levels, better subjective health status, higher weekly MVPA frequency, and longer MVPA duration are significantly associated with higher physical activity adherence. To ensure a more evidence-based discussion, it would be beneficial to focus on why these factors contribute to adherence to physical activity. For instance, individuals with higher education levels may have greater awareness of the benefits of physical activity, leading to stronger motivation for adherence. Similarly, those with better subjective health status may feel more capable of engaging in physical activity, reinforcing consistent participation [4]. Based on our findings, it is necessary to focus on the physical activity adherence of workers with lower education levels or poor subjective health status and to develop strategies to help them sustain their physical activity.

In this study, psychological need satisfaction in physical activity, a variable that directly affected physical activity adherence among office workers, was the factor that had the greatest direct impact. Moreover, it had a significant effect on physical activity adherence through the mediation of autonomous motivation. This finding is supported by previous studies, which reported that psychological need satisfaction in physical activity directly affected physical activity behaviors and significantly affected physical activity adherence through the mediation of autonomous motivation [21,39,40]. It is noted that psychological need satisfaction in physical activity involves autonomy (freely choosing the desired physical activity), competence (believing in one’s ability to perform the physical activity successfully), and relatedness (forming close relationships with others participating in the same physical activity), through which physical activity is sustained [12,31]. Our study findings demonstrated that physical activity should be sustained by enhancing three factors of PNSE (psychological need satisfaction in exercise) which have the greatest impact on sustaining physical activity. This is why there is a need to develop strategies to increase autonomy, competence, and relatedness for physical activity adherence among male office workers. Strategies to enhance autonomy can include providing not only options for individuals to choose the physical activity they like and prefer but also the time and place for such activity [19]. Moreover, since individuals perform physical activities better when provided immediate feedback, personalized feedback must be provided after a physical activity to improve competence in workers [19]. Finally, strategies to improve relatedness should include warm support among people participating in the same activity. These strategies must be applied to the physical activity program, since individuals find greater enjoyment and satisfaction through physical activity carried out in close relationships with several others [41].

As expected, autonomous motivation was a significant factor directly affecting physical activity adherence. This finding is supported by the self-determination theory and numerous previous studies that demonstrate autonomous motivation as a significant influencing factor for sustaining physical activity [10,13,21,39,42]. Autonomous motivation refers to the enjoyment, satisfaction, and interest perceived by individuals in MVPA itself, which causes them to actively adopt physical activity and sustain long-term activity [10,12,29]. Considering both the direct and mediating effects of autonomous motivation on physical activity adherence in this study, a strategy that promotes autonomous motivation is needed. For instance, several studies have reported the effectiveness of physical activity programs applying motivational coaching to increase enjoyment and satisfaction with physical activity or mobile health programs using motivational interviewing [43]. Therefore, workplace physical activity programs that apply these strategies must be implemented to promote autonomous motivation.

Autonomy support from significant others was a significant factor that had a direct effect on psychological need satisfaction in physical activity and an indirect effect on physical activity adherence through the mediation of psychological need satisfaction in physical activity and autonomous motivation. According to the SDT, autonomy support from significant others has a direct effect on basic psychological needs and an indirect effect on health behaviors through basic psychological needs and motivation [19]. Autonomy support from significant others not only promotes sustained health behaviors but also enhances personal growth [19]. Therefore, a strategy that enhances autonomy support must be implemented for physical activity adherence among participants so that significant others such as family or colleagues of participants can actively support their MVPA.

Contrary to expectations, perceived physical activity barriers did not have a direct effect on the physical activity adherence, but it had an indirect effect on physical activity adherence through psychological needs and autonomous motivation. In general, obstacles for discontinuing physical activity are lack of time, the high costs associated with MVPA, and inconvenient locations, while intrinsic factors of physical activity obstacles are lack of motivation, negative emotions toward regular physical activity, and insufficient information and skills [8,16]. According to previous research, such obstacles reduce the autonomy and competence related to physical activity adherence, thereby lowering activity motivation and hindering the sustained performance of MVPA [16]. Although our findings show that physical activity barriers do not have a direct effect on physical activity adherence, considering that many previous studies have found significant effects of these barriers on adherence, it is necessary to re-examine the relationship between them in future studies.

This study is significant in that it developed a structural equation model of physical activity adherence based on the SDT and identified complex causal relationships including direct and indirect effects for the physical activity adherence of office workers. Despite this significance, however, this study has limitations. First, participants in this study were male office workers, which makes it difficult to generalize the results to all male office workers. Second, a study with limited participants such as workers in small and medium banks may not be representative of all office workers because our participants would show significant differences in terms of organizational support for physical activity compared to workers in large banks or companies. Third, self-report data could be susceptible to social desirability bias, memory distortions, and potential for exaggeration or omission of information which can affect the reliability of research findings.

## 5. Conclusions

This study aimed to test the hypothesized relationships based on self-determination theory to investigate factors affecting the physical activity adherence of male office workers, and to provide information for developing workplace physical activity programs. Our results show that psychological need satisfaction in physical activity and autonomous motivation were significant variables that had direct effects on physical activity adherence, while autonomy support from significant others and perceived physical activity barriers had significant indirect effects through psychological need satisfaction and autonomous motivation, explaining 62.0%. Psychological need satisfaction in physical activity directly affected physical activity adherence, and the total effect was significant as it indirectly affected physical activity adherence through autonomous motivation. Autonomous motivation directly affected physical activity adherence. Autonomy support and perceived physical activity barriers were not significant direct effects on physical activity adherence, but the total effect was significant by indirectly affecting psychological need satisfaction in physical activity and autonomous motivation.

Based on the results of this study, the following suggestions can be made for further research. First, this study targeted male workers from small to medium banks with around 50 workers. Since the financial and environmental support for the physical activity adherence of workers may vary by company size, further research is needed involving male workers from larger banks or companies. Considering the increasing employment rate of female workers in the job market, further research is needed to investigate the factors influencing physical activity adherence among female workers, to examine the predictors of physical activity adherence, including both male and female workers, and to validate the moderating effects of gender as well. Second, it is necessary to develop workplace activity programs to improve the autonomy, competence, and relatedness aspects of psychological need satisfaction in physical activity. In addition, office workers could continue their physical activity if they recognize the social support from significant others and customized workplace-specific physical activity programs to promote autonomous motivation should be implemented.

## Figures and Tables

**Table 1:** Differences in Physical Activity Adherence by General Characteristics and Physical Activity-Related Characteristics of Participants.

Characteristics	Categories	*n* (%)	Physical Activity Adherence	
			M ± SD	t/F (*p*)
Age (year)	<30	27 (10.5)	2.35 ± 0.37	0.88 (0.454)
	30~39	86 (33.5)	2.23 ± 0.39	
	40–49	91 (15.4)	2.26 ± 0.37	
	≥50	53 (20.6)	2.30 ± 0.30	
	M ± SD	40.85 ± 8.52		
Marital status	Unmarried	176 (68.5)	2.28 ± 0.37	−0.69 (0.493)
	Married	81 (31.5)	2.24 ± 0.36	
Educational level †	High school ^a^	43 (16.7)	2.19 ± 0.58	5.34 (0.005)
	College ^b^	56 (21.8)	2.16 ± 0.36	b < c
	University ^c^	158 (61.5)	2.32 ± 0.35	
Having a chronic disease	Yes	59 (23.0)	2.26 ± 0.36	−0.14 (0.886)
	No	198 (77.0)	2.27 ± 0.37	
Subjective health status †	Poor ^a^	17 (6.6)	2.01 ± 0.38	7.83 (0.001)
	Moderate ^b^	80 (31.1)	2.20 ± 0.35	a < c
	Good ^c^	160 (62.3)	2.33 ± 0.36	
Total working experience	<10	110 (42.8)	2.27 ± 0.37	0.93 (0.395)
(years)	10–19	80 (31.1)	2.23 ± 0.40	
	≥20	67 (26.1)	2.31 ± 0.31	
	M ± SD	12.39 ± 8.94		
Amount of MVPA	30–59	28 (34.6)	2.21 ± 0.34	0.35 (0.705)
(min/day)	60–89	104 (32.3)	2.27 ± 0.34	
	≥90	125 (33.1)	2.27 ± 0.39	
	M ± SD	85.21 ± 37.96		
Frequency of MVPA	3	124 (48.2)	2.21 ± 0.39	−2.52 (0.012)
(days/week)	4–7	133 (51.8)	2.32 ± 0.34	
	M ± SD	3.75 ± 1.23		
Years of MVPA duration †	<3 ^a^	152 (59.2)	2.18 ± 0.36	11.87 (<0.001)
	3–5 ^b^	36 (14.0)	2.29 ± 0.31	a < c
	>5 ^c^	69 (26.8)	2.43 ± 0.36	
	M ± SD	4.17 ± 5.47		
Physical activity adherence †	Adherence unlikely ^a^	115 (44.7)	-	-
	Adherence possible ^b^	113 (44.0)	-	-
	Adherence likely ^c^	29 (11.3)	-	-

Note: † Scheffé post hoc test.

**Table 2:** Descriptive Statistics and Convergent Validity of Variables.

Variables	Range	M ± SD	Skewness	Kurtosis	β	CR	AVE
Perceived physical activity barriers	1–4	1.69 ± 0.38	0.09	0.58	-	-	-
Autonomy support	1–5	3.71 ± 0.62	−0.45	0.12	-	-	-
Psychological need satisfaction in physical activity	1–6	4.64 ± 0.74	−0.74	0.92	-	0.84	0.63
Autonomy		4.88 ± 0.75	−0.98	1.86	0.63	-	-
Competence		4.40 ± 1.01	−0.62	0.17	0.81	-	-
Relatedness		4.65 ± 0.87	−1.03	2.08	0.81	-	-
Autonomous motivation	0–4	3.20 ± 0.56	−0.78	0.99	-	-	-
Physical activity adherence	12–36	27.19 ± 4.39	−0.19	−0.30	-	0.95	0.87
Predisposing		9.43 ± 1.84	−0.26	−0.58	0.84	-	-
Enabling		9.11 ± 1.77	−0.05	−0.75	0.79	-	-
Reinforcing		8.28 ± 1.82	−0.02	−0.30	0.66	-	-

Note: SD = standard deviation; CR = construct reliability; AVE = average variance extracted.

**Table 3:** Direct, Indirect, and Total Effects of the Hypothetical Model.

Endogenous Variables	Exogeneous Variables	Parameter Statistics		Direct Effect	Indirect Effect	Total Effect	SMC
		SE	C.R.	β (*p*)	β (*p*)	β (*p*)	
Psychological need satisfaction in physical activity	Autonomy support	0.44	6.67	0.44 (<0.001)	-	0.44 (<0.001)	0.46
	Perceived physical activity barriers	−0.42	−6.45	−0.42 (<0.001)	-	−0.42 (<0.001)	
Autonomous motivation	Autonomy support	0.01	0.18	0.01 (0.861)	0.29 (0.011)	0.30 (0.007)	0.51
	Perceived physical activity barriers	−0.09	−1.58	−0.09 (0.115)	−0.28 (0.005)	−0.37 (0.007)	
	Psychological need satisfaction in physical activity	0.66	7.02	0.66 (<0.001)		0.66 (<0.001)	
Physical activity adherence	Autonomy support	0.03	0.39	0.03 (0.695)	0.33 (0.005)	0.36 (0.011)	0.62
	Perceived physical activity barriers	0.02	−0.35	0.02 (0.723)	−0.34 (0.002)	−0.32 (0.014)	
	Psychological need satisfaction in physical activity	0.52	4.35	0.52 (<0.001)	0.22 (0.004)	0.74 (0.005)	
	Autonomous motivation	0.33	3.93	0.33 (<0.001)	-	0.33 (<0.001)	

Note: SE = standardized estimates; C.R. = construct reliability; SMC = squared multiple correlations.

## Data Availability

The datasets generated and analyzed during the current study are not publicly available due to concerns regarding participant privacy. The data presented in the study can be available from the corresponding author upon reasonable request.
